# Reversible Low-Light Induced Photoswitching of Crowned Spiropyran-DO3A Complexed with Gadolinium(III) Ions 

**DOI:** 10.3390/molecules17066605

**Published:** 2012-05-31

**Authors:** Klaus Kruttwig, Diego R. Yankelevich, Chantal Brueggemann, Chuqiao Tu, Noelle L’Etoile, André Knoesen, Angelique Y. Louie

**Affiliations:** 1 Department of Biomedical Engineering, University of California, Davis, CA 95616, USA; 2 Department of Electrical and Computer Engineering, University of California, Davis, CA 95616, USA; 3 Department of Cell and Tissue Biology, University of California, San Francisco, CA 94143, USA

**Keywords:** spiropyran, photochromic molecule, DO3A, MRI contrast agent, luciferase, light emitting diodes, rate constant, low light applications

## Abstract

Photoswitchable spiropyran has been conjugated to the crowned ring system DO3A, which improves its solubility in dipolar and polar media and stabilizes the merocyanine isomer. Adding the lanthanide ion gadolinium(III) to the macrocyclic ring system leads to a photoresponsive magnetic resonance imaging contrast agent that displays an increased spin-lattice relaxation time (*T*_1_) upon visible light stimulation. In this work, the photoresponse of this photochromic molecule to weak light illumination using blue and green light emitting diodes was investigated, simulating the emission spectra from bioluminescent enzymes. Photon emission rate of the light emitting diodes was changed, from 1.75 × 10^16^ photons·s^−1^ to 2.37 × 10^12^ photons·s^−1^. We observed a consistent visible light-induced isomerization of the merocyanine to the spiropyran form with photon fluxes as low as 2.37 × 10^12^ photons·s^−1^ resulting in a relaxivity change of the compound. This demonstrates the potential for use of the described imaging probes in low light level applications such as sensing bioluminescence enzyme activity. The isomerization behavior of gadolinium(III)-ion complexed and non-complexed spiropyran-DO3A was analyzed in water and ethanol solution in response to low light illumination and compared to the emitted photon emission rate from over-expressed *Gaussia princeps* luciferase.

## 1. Introduction

Spiropyrans represent the most widely studied class of organic photochromes, but the exact mechanism of photocoloration has yet to be determined [1]. In general, spiropyran (SP) is stable in its closed-ring isomeric form, and is a colorless or pale yellow solution in non-polar solvents. After exposure to UV irradiation, this SP form is converted to a metastable open-ring isomer (merocyanine, MC), possessing an optical absorption peak at 550–600 nm. The original colorless SP form, can be restored via visible light irradiation and/or thermal induction. Conjugation of a nitrogroup into the benzene ring of the chromene part of spiropyran acts as a π-accepting substituent [2]. This leads to a strong bathochromic shift in the open-chain isomer [1]. Spiropyrans in their closed form are soluble in a wide range of organic solvents and display quite low water solubility [3]. This impairs/restricts their use in cellular biological applications, where water solubility is required. In fact, spiropyran isomerization and effects of substitutions on the isomerization have been well investigated in organic solvents, whereas little is known about those in aqueous solution [4]. 

Water-soluble spiropyrans have been synthesized by the introduction of a sulfonate group on the phenyl ring or 8-methoxy-6-nitro-BIPS [5]. The conjugation of a crown ether moiety is considered necessary to stabilize the merocyanine form through metal ion complexion formation and to produce a polar environment, inducing similar properties as those found with the application of polar solvents [6]. Substitution of spiropyrans with monoaza-15-crown-5, 15-crown-5 fragments or acyclic analogs in position 6’ or the nitrogen atom of the indoline ring, as well as in position 8’ of the benzopyrene part has a large influence on the photochromic properties of the molecule [7]. It has been reported that a crowned metal cation is capable of forming a coordination bond with the phenolate oxygen anion, which eventually leads to the stabilization of the merocyanine form. Photoirradiation of crown-containing spiropyrans leads to a transformation into the closed form of the molecule with a subsequent release of the metal ion. 

**Scheme 1:**
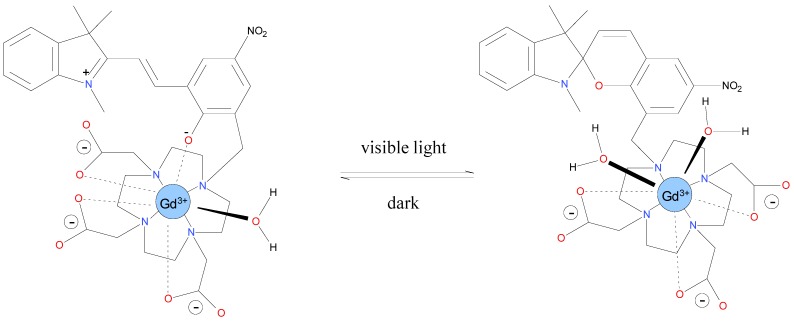
Structural representation of spiropyran-DO3A-Gd(III): Proposed isomerization of the compound, displaying the two conformational states: merocyanine (left) and spiropyran (right). Under dark conditions, spiropyran-DO3A is preferentially in the merocyanine (MC) form, whereas visible light illumination leads to isomerization to the spiropyran form (SP).

Previously we have reported the conjugation of spiropyran to a DO3A-macrocycle and further complexion of a gadolinium ion (Gd^3+^ ion) and successful application as light responsive Magnetic Resonance Imaging (MRI) contrast agent (Scheme 1) [2,8]. Due to the electrostatic interaction of the complexed metal ion with the indoline part of the photochromic molecule, the hydration state of Gd(III) is altered depending on the isomerization state of spiropyran, resulting in a modification of the contrast agent relaxivity. Photochromism of spiropyrans is affected by several factors, such as the temperature, the nature of the solvent and the substitution of side groups. The light power density (irradiance) that has been reported required to initiate a photochromic conversion ranges from below 0.9 mW·cm^−2^ up to several 100 mW·cm^−2^, but most of the reports in the literature are lacking precise photometric details [9,10,11,12,13,14,15,16].

Recently, studies have demonstrated that low power irradiation using light emitting diodes (LEDs, with an irradiance of approximately 1 mW·cm^−2^) can be used to effectively trigger the SP to MC isomerization [9,17]. In fact, Yoshida *et al.* demonstrated a linear correlation between switching time and laser power in spiropyran-doped film [18]. They reported a maximum response time of 200–300 ms with a power of approx. 50 mW of the laser at λ = 532 nm. But, the rate constants and irradiance associated with these results were not reported [18]. To our knowledge, there have been no systematic studies reported that analyze the effect of low light illumination on spiropyran-based molecules possessing a photostable MC state. Measuring the amount of light capable of inducing photochromic conversions is of particular importance for experimental studies where light levels are restricted, such as in biological tissue applications. There, the visible light penetration depth is limited, dependent on the wavelength, the absorption, and scattering properties of the tissue [19,20]. This limited light penetration would seem to preclude the *in vivo* application of light switchable molecules, which have great potential as pharmaceutical and imaging systems. Examples of the use of spiropyrans *in vivo* have been reported, with some degree of success. For example, Ipe *et al.* described a spiropyran-based drug release system, where the amino acid derivative L-3,4-dihydroxyphenylalanine (L-DOPA) could be released upon light stimulation [21]. Additionally, an antibody-mediated targeting of a spiropyran containing a two-photon imaging probe to breast cancer cells *in vitro* has been recently described [22]. These are key developments for future *in vivo* studies for selective tumor cell labeling, light induced release of pharmaceutics, and the application of contrast agents for non-invasive imaging. These studies clearly demonstrate the emerging potential of reversible photochromic molecules in diagnostic and therapeutic applications. 

Our group has previously described the synthesis and proof-of-principle application for a DO3A conjugated spiropyran and dinitrospiropyran to be used as a reversible MRI imaging probe *in vivo* [2,8]. Understanding both the photochromic behavior and the relaxivity properties (e.g., the spin-lattice relaxation time *T*_1_, *etc.*) of spiropyran-based probes in response to light irradiation is necessary for advancing its biological applications. Herein, we describe our investigation of the switching behavior of spiropyran-DO3A using defined spectral irradiation properties, mimicking emission from a bioluminescent enzyme with low power illumination in different solvents. Emission of a bioluminescence signal refers to the generation of visible light from an enzyme-catalyzed reaction of molecular oxygen with the substrate luciferin [23]. Firefly luciferase (FLuc) from *Photinus pyralis* is one of the most commonly used luciferases in applications of bioluminescence, exhibiting a broad spectral emission with a peak at 562 nm [24,25]. The click beetle luciferase from *Pyrearinus termitilluminans*, displays a maximum light emission at λ = 538 nm and the highest quantum yield reported yet of 0.61 [26,27]. Alternatively, ATP-independent luciferases, using coelenterazine as a substrate like *Gaussia princeps* luciferase (GLuc) with a peak light emission at 470 nm can be used [28]. A codon-optimized variant with a 200 fold (*in vivo*) to 1000 fold (*in vitro*) higher bioluminescence signal intensity when compared to FLuc and *Renilla* luciferase (RLuc) will be used in our study to compare LED emitted photon flux with enzymatically produced light [29,30]. Overall, we describe a novel system in which spiropyran tethered MRI contrast agents respond to low light such that MRI may be used to map the expression of bioluminescent markers. In addition, we report the properties of previously developed set of novel light responsive MRI probes that can be reversibly activated by illumination with visible light [2,8].

## 2. Results and Discussion

### 2.1. Spectroscopic Characteristics of Gd(III) Complexed and Non-Complexed Spiropyran-DO3A

**Figure 1:**
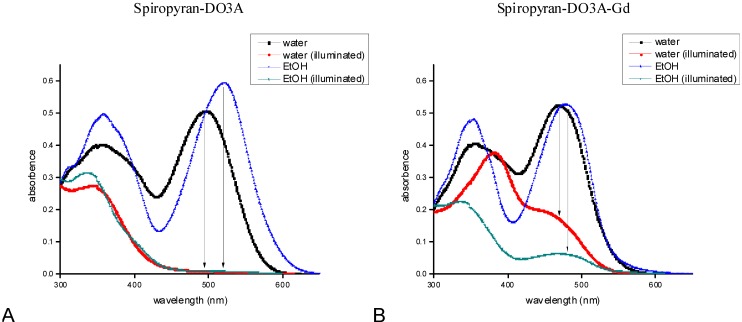
Spectroscopic characteristics of spiropyran-DO3A complexed and non-complexed with Gd(III): Absorption of water and ethanol solutions of spiropyran-DO3A were spectroscopically analyzed (**A**). Illumination of the solutions leads to a decrease in absorption and a shift of the actinic band in the range between 350 nm and 400 nm. A shift in the peak absorption wavelength of spiropyran-DO3A complexed with Gd(III) ions in ethanol and water could be detected (**B**). Illumination of spiropyran-DO3A-Gd in water leads to a decreased MC to SP conversion compared with the ethanol solution. Illumination was performed with a current of 18.5 mA, corresponding to 1.754 × 10^16^ photons·s^−1^.

Peak absorbance values for the crowned spiropyran and crowned spiropyran complexed with Gd(III) ions were adjusted to 0.5–0.6 in water and 0.6–0.7 in EtOH, respectively. Spiropyran-DO3A dissolved in water displayed two peak absorption bands, one in the region between λ = 300 nm to 400 nm with a maximum at 350 nm and one from 400 nm to 550 nm displaying a peak absorbance of 0.514 (±0.011) at 496 nm (Figure 1A). A red shift and increase of the peak absorption peak to 0.606 (±0.02) at 520 nm was observed when the contrast agent was dissolved in ethanol solution (Figure 1A).

In contrast to spiropyrans bearing free hydroxy, carboxy or amino groups either on the indoline or the benzopyran part, which exhibit negative photochromism, the DO3A tethered spiropyran displayed positive photochromism in both the solvents water and ethanol [5]. Illumination for 1 min with the blue LED and a total photon emission rate of 1.754 × 10^16^ photons·s^−1^ resulted in a significant decrease of the absorbance values from 0.514 (±0.011) to 0.01 (±6.82 × 10^−4^) in the water solution, which represents an illumination-induced change of 98.05%. A slightly larger absorbance decrease of 98.5% was detected after illumination ofspiropyran-DO3A in ethanol solution.

The Gd-complexed spiropyran-DO3A molecule displayed a peak absorption peak of 0.519 (±0.012) at 470 nm in water solution, which decreased to 0.165 (±0.012) after illumination (Figure 1B). The observed values are in accordance to the values reported previously by Tu *et al.* [8]. Dissolving spiropyran-DO3A-Gd in EtOH leads to a red-shifted peak of 0.547 (±0.019) at 478 nm, which decreases to 0.066 (±4.28 × 10^−3^) after illumination (Figure 1B). A shift of the absorbance dependent on the surrounding environment, as observed in the current study, was described previously [31]. Interestingly, the observed hypsochromic wavelength shift, while increasing the polarity of the surrounding environment from EtOH to water, was three times larger when comparing non-complexed and Gd-complexed spiropyran-DO3A. The observed, photostationary stable merocyanine conformation has been described for crowned spirobenzopyrans earlier and may have its foundation in a strong electrostatic interaction between the high charge density of the phenoxide oxygen and the cyclen-complexed gadolinium cation [8]. 

The length of the alkyl chain is one of the factors that influence the interaction between the metal ion and the photochromic part of the molecule [7]. Tethering of the DO3A macro complex to the photochromic molecule was achieved in the present investigation through a one carbon linker molecule (Scheme 1). The length of this linker has a pivotal role on switching efficiency, was previously shown by Diamond *et al.* using a covalently functionalized polystyrene bead-based system [17]. Varying the linker length from four to eight carbon atoms significantly improved the photochromic conversion of this polystyrene-bead-conjugated spiropyran [17].

### 2.2. Correlation between Rate Constants and Total Photon Emission Rate

**Figure 2:**
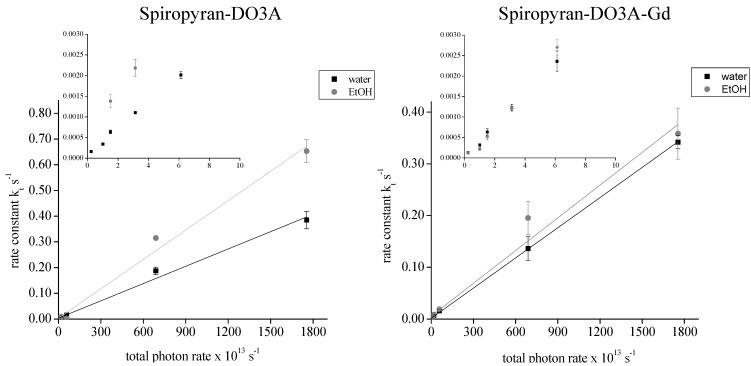
Determination of rate constants and maximum MC to SP conversion in relation to the total photon emission rate: The rate constants of spiropyran-DO3A-Gd, dissolved in water and ethanol were determined after illumination with the blue LED using equation (2). The insert displays the data points between 0 s^−1^ and 10 × 10^13^ s^−1^. A linear regression analysis was performed which gave R^2^ = 0.99156 for spiropyran-DO3A dissolved in water and R^2^ = 0.98982 for spiropyran-DO3A dissolved in ethanol. For spiropyran-DO3A-Gd dissolved in water the regression analysis gave R^2^ = 0.99978 and R^2^ = 0.97866 for spiropyran-DO3A-Gd dissolved in EtOH.

Photokinetic analysis can be applied in the case of spiropyran-DO3A conjugated molecules, where thermal isomerization accompanies the photochemical processes and the rate of photostationary conversion depends on the irradiation wavelength and on the intensity of the incident photon flux [32]. For determination of rate constants for the light-induced MC to SP conversion of Gd(III), complexed and non-complexed spiropyran-DO3A, dissolved in water and/or ethanol, were illuminated for defined time periods. Spiropyran-DO3A was stable in the merocyanine form when dissolved in water and kept in the dark. It displayed absorbance fluctuations of approximately 5%, but no gradual decrease of absorbance could be detected in contrast to spiropyran-DO3A dissolved in EtOH. In EtOH, a decrease in absorbance values with a rate constant of 1.0 × 10^−3^ s^−1^ was calculated under control (dark) conditions, clearly displaying a decreased half-life of the photostationary merocyanine state in EtOH as compared to water solution. Illumination of spiropyran-DO3A, dissolved in water with photon fluxes between 1.754 × 10^16^ photons·s^−1^ and 2.37 × 10^12^ photons·s^−1^ induced a merocyanine to spiropyran isomerization. The light induced MC to SP isomerization process followed first order kinetics, as has been previously described [33]. The calculated rate constants were between k_t_ = 0.385 (±3.4 × 10^−2^) s^−1^ and k_t_ = 1.61 × 10^−4^ (±2.25 × 10^−5^) s^−1^, displaying a linear regression coefficient of R^2^ = 0.99156 when dissolved in water and a linear regression coefficient of R^2 ^= 0.98982 when dissolved in ethanol (Figure 2A). Illumination of spiropyran-DO3A dissolved in EtOH leads to significant higher rate constants as compared to the condition when the same molecule was dissolved in water. For the MC to SP isomerization of spiropyran-DO3A in ethanol under continuous illumination with 1.754 × 10^16^ photons s^−1^, the rate constant was k_t_ = 0.653 (±4.5 × 10^−2^) s^−1^. At the lowest photon count tested, irradiation with 1.50 × 10^13^ photons·s^−1^, a rate constant was k_t_ = 1.379 × 10^−3^ (±1.56 × 10^−4^) s^−1^. Because of the unstable MC state, we have not applied lower photon fluxes. It is noteworthy that spiropyran-DO3A possesses a maximal absorbance at 520 nm, while the maximum emission of the blue LED is at 465 nm. This implies that a higher rate constant can be achieved after ideal matching of the emission spectrum and the contrast agent absorbance spectrum, which we will be shown for the green LED in section 3.6.

The complexion of spiropyran-DO3A with Gd(III) ions leads to a superior stabilization of the merocyanine form. Under control (dark) conditions, fluctuations in absorbance values of 1.46% in water solution and 3.24% in ethanol solution have been observed. In contrast to spiropyran-DO3 dissolved in EtOH, no gradual decrease in absorbance could be detected under dark conditions. Illumination of spiropyran-DO3A-Gd dissolved in water with total photon rates between 1.754 × 10^16^ photons·s^−1^ and 2.37 × 10^12^ photons·s^−1^ induced a merocyanine to spiropyran isomerization with calculated rate constants between k_t_ = 0.3266 (±0.034) s^−1^ and k_t_= 1.28 × 10^−4^ (±1.89 × 10^−5^) s^−1^ and a linear regression coefficient of R^2^ = 0.99978 (Figure 2B). Illumination of spiropyran-DO3A-Gd, dissolved in EtOH does not lead to significantly higher rate constants compared to the water solution as observed for the non-complexed contrast agent. For the MC to SP isomerization under continuous illumination with 1.754 × 10^16^ photons·s^−1^, the rate constant was k_t_ = 0.358 (±0.049) s^−1^. Illumination of the ethanol solution with 2.37 × 10^12^ photons·s^−1^ induced a merocyanine to spiropyran conversion with k_t_ = 1.21 × 10^−4^ (±1.21 × 10^−5^) s^−1^. The linear regression coefficient was R^2^ = 0.97866. We were able to induce a MC to SP isomerization applying photon emission rates below the sensitivity range of the used optical power meter (<2.37 × 10^12^ photons·s^−1^) with spiropyran-DO3A-Gd dissolved in water, but not when dissolved in EtOH. While further decreasing the total photon emission rate with applying a current of 0.014 mA lead to a rate constant of 3.31 × 10^−5^ (± 5.40 × 10^−6^) s^−1^ in water, no MC to SP conversion was inducible in ethanol solution at this photon density. 

**Figure 3:**
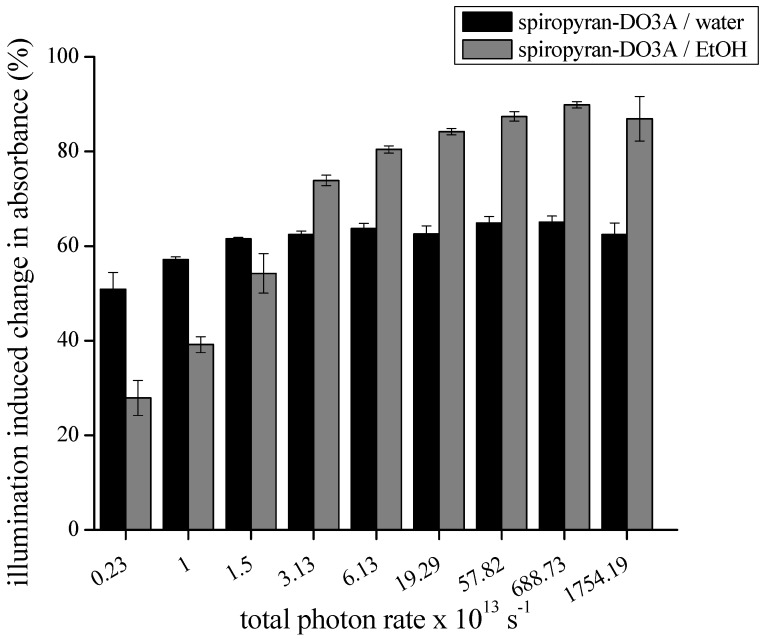
Determination of maximum illumination-induced peak absorbance change in relation to the photon emission rate: The maximum peak absorbance change of spiropyran-DO3A-Gd dissolved in water and ethanol for different photon emission rates intensities was determined after illumination with the blue LED. The total photon emission rate was calculated using Equation (3).

To answer the question of what percentage of the spiropyran containing molecules can be efficiently interconverted to the spiropyran state at a defined photon emission rate, spiropyran-DO3A-Gd and spiropyran-DO3A in water or ethanol solution were illuminated until no further conversion was observed (Figure 3). Illumination of spiropyran-DO3A-Gd in water with a total photon emission rate of 1.754 × 10^16^ photons·s^−1^ leads to a change in absorbance of 62.46 (±2.44) % whereas a isomerization of 86.95 (±4.71) % could be induced in ethanol solution.

Decreasing the total photon rate leads to a decreased change in absorbance and a further extension of the illumination time needed for the interconversion. This impairment was significantly greater in ethanol solution compared with spiropyran-DO3A-Gd dissolved in water. Illumination with 2.37 × 10^12^ photons·s^−1^ lead to peak absorbance change of 50.86 (±3.56) % when dissolved in water. This is in contrast to a peak absorbance change of 27.9 (±3.72) % when dissolved in ethanol. At photon rates below 2.37 × 10^12^ photons·s^−1^ the peak absorbance changed markedly in water solution to 34.79 (±2.45) %, and no change was observed in ethanol solution (data not shown in Figure 3). To calculate the rate of the thermally-induced back-reaction after continuous illumination, we measured a 12-fold increase in the rate constant of spiropyran-DO3A when dissolved in ethanol *versus* water (Figure 3) at comparable absorbance values.

### 2.3. Investigation of Increased Temperature on Back-Conversion and Repeated Switching Cycles

**Figure 4:**
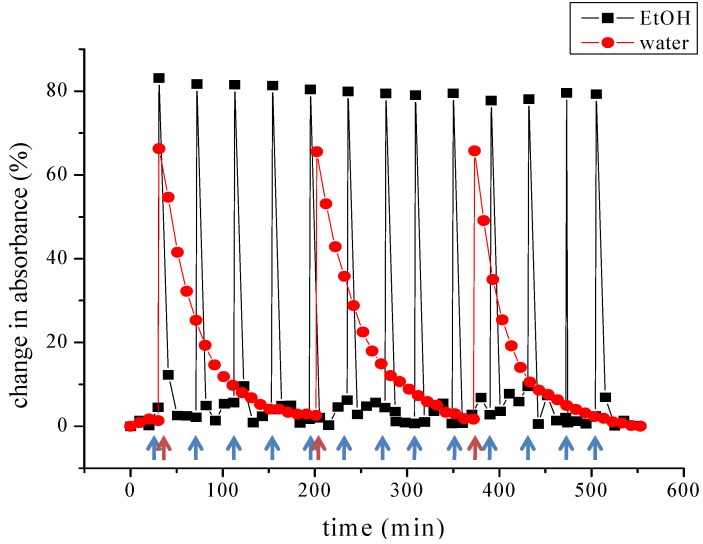
Reversible interconversion between the spiropyran and the open merocyanine form: The thermal-induced back-reaction from the SP form to the MC form of spiropyran-DO3A-Gd in water and ethanol solution was determined after illumination at a temperature of 37 °C. Blue arrows represent the irradiation time points of the water solution, while red arrows represent illumination of spiropyran-DO3A-Gd in ethanol.

Photodegradation of the merocyanine form of the spiropyran molecule is a well-known problem that limits the application of reversible, self-regenerating sensing molecules [34]. We focused further on describing the photochemical behavior of spiropyran-DO3A-Gd at physiologically relevant temperatures. Increasing the temperature from 25 °C to 37 °C had no influence on the reaction kinetics observed while continuously illuminating spiropyran-DO3A-Gd dissolved in water. Also, the absorption spectra of the merocyanine form did not change at 37 °C during the course of the investigation, which is in accordance with the description from Movia *et al.* [35] In contrast to these observations while illuminating the sample, the thermally-induced back-reaction was affected by temperature. After terminating the irradiation for 1 min with a total photon emission rate of 1.754 × 10^16^ photons·s^−1^ the thermal relaxation rate was determined at 25 °C to be k_t_ = 1.1 × 10^−4^ (±1.3 × 10^−4^) s^−1^. This rate constant increased to k_t_ = 5.04 × 10^−4^ (±7.29 × 10^−4^) s^−1^ at the temperature of 37 °C. We then investigated the influence of repeated switching cycles on the MC to SP conversion (Figure 4). When dissolved in water, switching of spiropyran-DO3A-Gd could be induced at least 3 times without impairment of complete restoration of the merocyanine form at 37 °C. We focused on the thermodynamically induced interconversion to the merocyanine state instead of using UV illumination, which is known to induce photodegradation [17]. Also, for potential *in vivo* application of spiropyran-DO3A-Gd, it is critical to ensure the complete restoration of the merocyanine form without additional illumination. The time needed for complete restoration of the MC form was calculated to be approximately 174 min. The rate constant for the thermal-induced back-reaction of spiropyran-DO3A-Gd in ethanol solution increased from 5.67 × 10^−3^ (±4.16 × 10^−3^) s^−1^, determined at room temperature 1.52 × 10^−2^ (±1.20 × 10^−3^) s^−1^ at 37 °C. The time needed for complete restoration of the MC form was calculated to be approximately 40 min, when dissolved in EtOH. 

Our results, obtained with the application of several interconversion cycles between the spiropyran form and the merocyanine form, also clearly displayed that the amount of photobleaching is greatly reduced using low power LED illumination with 1.754 × 10^16^ photons·s^−1^ which has also been previously reported by Scarmagnani *et al.* [17].

### 2.4. Relaxometric Properties

**Figure 5:**
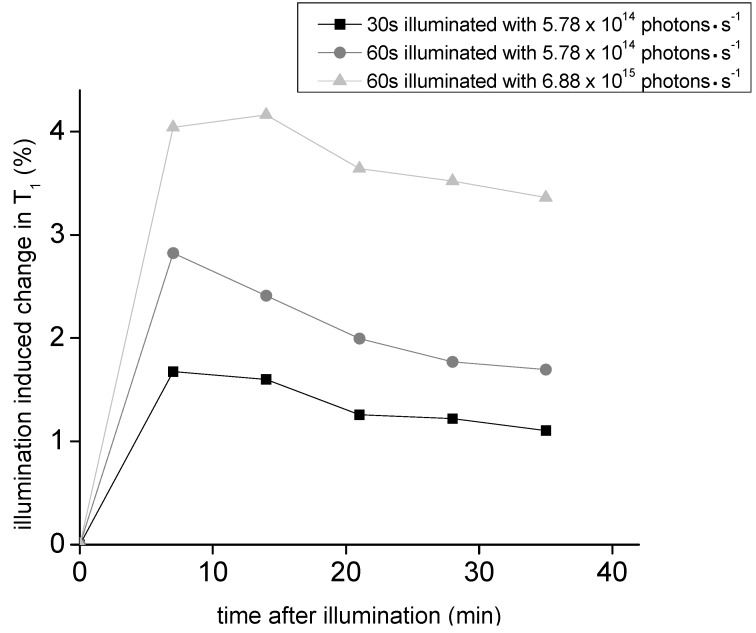
Relaxometric properties of spiropyran-DO3A-Gd in water: Determination of the relaxometric properties in response to light stimulation was performed with spiropyran-DO3A-Gd in water solution after brief light pulses.

Relaxometric properties of spiropyran-DO3A-Gd were analyzed using concentrations between 39.4 μM and 157.6 μM. Spiropyran-DO3A-Gd kept under dark conditions possesses an *r*_1_ of 2.93, whereas the *r*_1_ value decreased to 2.63 after illumination for 60 s with the blue LED using a photon emission rate of 1.754 × 10^16^ photons·s^−1^. This represents a light-induced change in relaxivity of 10.24%. The interaction of water molecules with the first coordination sphere of the Gd-containing contrast agents likely produces changes in the *T*_1_ properties of the CA. The decrease in the *r*_1 _relaxivity of the contrast agent upon visible light illumination may reflect the structural distinction between the merocyanine and spiropyran isomeric forms. In the spiropyran form, the complexed Gd(III) may be attracted to the indoline part of the photochromic molecule where electrostatic interactions between the metal ion and non-bonding electrons take place [2]. Thus, the indoline part influences water molecule access to the Gd(III) ion and impairs *T*_1_ contrast generation. Experimentally, spiropyran-DO3A-Gd, dissolved in water was illuminated for different time periods with the blue LED, immediately followed by *T*_1_ determination using relaxometry (Figure 5). The Δ*T*_1_ values obtained after applying different illumination intervals corresponds to the observed absorbance changes, determined with absorbance spectroscopy. The largest change in *T*_1_ could be observed after 1 min irradiation with a total photon emission rate of 6.88 × 10^15^ photons·s^−1^.

### 2.5. Illumination with Two Different LED’s

Considering the differences in the peak absorption bands of non-complexed and complexed spiropyran-DO3A in different solvents, illumination with LEDs possessing the maximal emission at either 465 nm or 525 nm were performed. Although, clearly, matching the peak absorption band of spiropyran-DO3A and spiropyran-DO3A-Gd to the emission wavelength of the LED would give maximal response, we also sought to determine if the CA could respond to luciferase systems that only partially overlapped the excitation maximum. 

It has been reported that spiropyran is most sensitive in the green spectrum, compared to illumination with a red LED [9]. The adjustment of both LEDs to an identical photon emission rate of 5.78 × 10^14^ photons·s^−1^ enables a comparative measurement. Rate constants were determined for complexed and non-complexed spiropyran-DO3A dissolved in water and ethanol after illumination using the two LEDs (Table 1). The data clearly shows, when comparing the contrast agents either dissolved in EtOH or water that increasing the distance between absorbance peak of the CA and the maximum emission of the light source significantly lowers the rate constants. However, the less effective blue emission is still able to switch the CA even at low photon densities. 

**Table 1:** Illumination with two LEDs: Spiropyran-DO3A and spiropyran-DO3A-Gd were illuminated with green LED possessing maximum emission at λ = 525 nm and the blue LED (λ = 465 nm). The illumination power was equally adjusted to a total photon flux of 5.78 × 10^14^ photons·s^−1^. * The column Δmax [nm] refers to the separation between the absorption peak of the CA in relation to the maximum emission peak of the LED.

LED emission max	Solvent	Δmax (nm)	Spiropyran-DO3A rate constant	Δmax (nm)	Spiropyran-DO3A-Gd rate constant
465 nm	Water	31	1.55 × 10^−2^ ± (2.38 × 10^−3^) s^−1^	5	1.59 × 10^−2^ ± (1.99 × 10^−3^) s^−1^
	EtOH	53	2.29 × 10^−2^ ± (1.81 × 10^−3^) s^−1^	13	1.89 × 10^−2^ ± (3.51 × 10^−4^) s^−1^
525 nm	Water	29	1.29 × 10^−3^ ± (3.68 × 10^−3^) s^−1^	55	6.27 × 10^−3^ ± (4.76 × 10^−4^) s^−1^
	EtOH	7	1.94 × 10^−2^ ± (7.02 × 10^−4^) s^−1^	47	8.31 × 10^−3^ ± (1.50 × 10^−3^) s^−1^

### 2.6. Overexpression of Gaussia Princeps Luciferase

**Figure 6:**
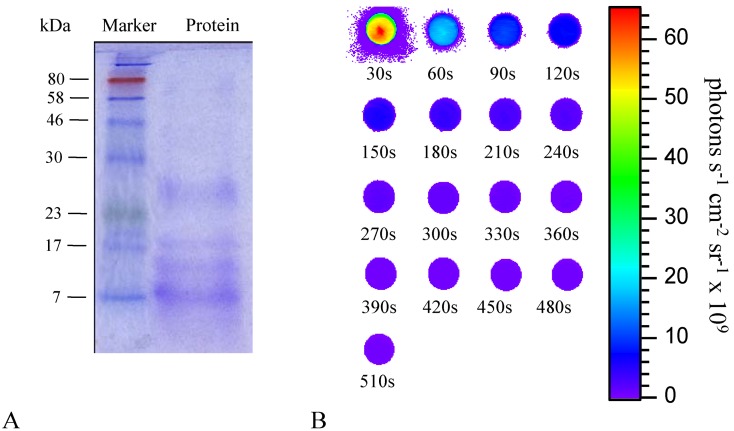
Optical properties of overexpressed *Gaussia princeps* luciferase: *Gaussia princeps* luciferase was overexpressed in a bacterial expression system after induction with IPTG and purified using affinity chromatography. SDS-polyacrylamide electrophoresis after Ni-IDA purification displays an enriched protein amount (**A**). 1.36 μg total protein amount was used for analysis. Determination of total photon flux was performed in an IVIS-100 imaging system over a time period of 510 s after supplementation of coelenterazine (**B**). Quantification of the total photon flux of the spots is shown in Figure 7.

**Figure 7:**
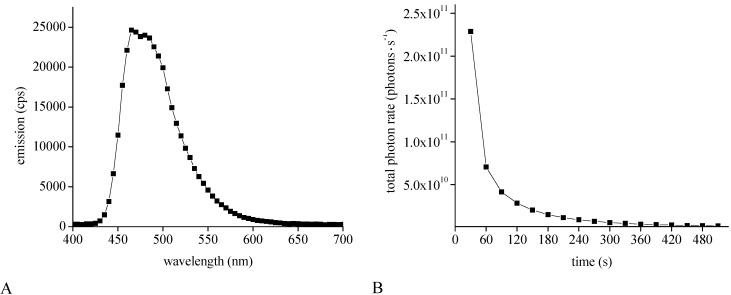
Optical properties of overexpressed *Gaussia princeps* luciferase: *Gaussia princeps* luciferase was overexpressed in a bacterial expression system after induction with IPTG and purified using affinity chromatography. Analysis of the emission spectrum displays the maximum intensity at 465 nm (**A**). Determination of total photon emission rate was performed in an IVIS-100 imaging system over a time period of 510 s (**B**).

**Figure 8:**
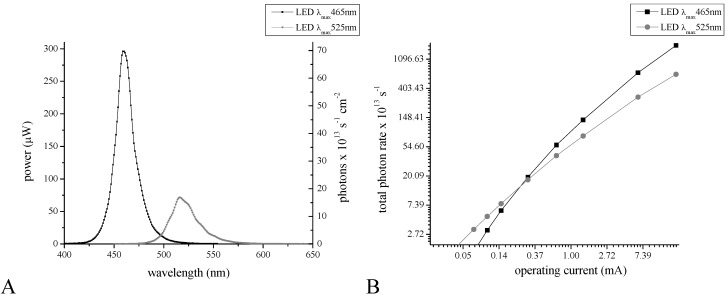
Spectral characteristics of LEDs: The spectral characteristics of the blue-emitting LED λ_max_ = 465 nm and the green emitting LED λ_max_ = 525 nm were recorded at the operating current of 18.5 mA (**A**). Operating current *versus* photon emission rate is displayed in (**B**).

After successful determination of photon emission rate and illumination time needed for inducing isomerization of the merocyanine to the spiropyran form, we evaluated the photon emission rate of the enzyme *Gaussia princeps* luciferase to validate if the application of light producing enzymes can induce an isomerization of the contrast agent. Overexpression of luciferases in eukaryotic cells also allows for high photon production. Values up to 5.2 × 10^4^ photons·s^−1^ for a single firefly luciferase overexpressing tumor cell have been described in previous literature [36]. After *in vivo* transplantation of luciferase overexpressing cells and the formation of tumors *in vivo*, values up to 1 × 10^11^ photons·s^−1^ could be shown using whole body imaging systems [36,37]. Recently it has been shown that *Gaussia* luciferase, isolated from the marine copepod *Gaussia princeps* emits brighter light than FLuc [30,38]. Therefore, we focused on this enzyme. N-terminal tagged *Gaussia princeps* luciferase was overexpressed under the T7 promoter using a bacterial expression system and purified with Ni-IDA affinity chromatography. After purification, SDS-polyacrylamide electrophoresis was performed, verifying a protein band at approximately 20 kDa, corresponding to the *Gaussia princeps* luciferase with a molecular weight of 19.9 kDa (Figure 6A) [30]. Spectral analysis confirmed a maximum emission at 465 nm with a broad emission spectrum extending to 600 nm after supplementation of coelenterazine (Figure 7A), as reported previously [30]. The maximum emission wavelength corresponds to the maximum emission wavelength of the blue LED (Figure 8A). Using the optical imaging system IVIS-200 we recorded the emission kinetics of the bioluminescence reaction after supplementation of the substrate coelenterazine.

Thirty s after substrate addition we observed a bright light emission with a total photon flux of 2.289 × 10^11^ photons·s^−1^ (Figure 6B and Figure 7B). Light emission decreased rapidly in the following 60 s to 2.855 × 10^10^ photons·s^−1^. Purified GLuc exhibits flash kinetics, which decays rapidly over time in contrast to the enzyme firefly luciferase, which has glow kinetics [30]. Verhaegen *et al.* reported a light emission peak at 1 s after substrate addition followed by a rapid decline, dependent on the coelenterazine concentration [39].

Producing appreciable quantities of properly folded GLuc has been reported to be difficult, primarily because of the presence of multiple disulfide bonds [40]. Goerke *et al.* showed that 5 μg·mL^−1^ of GLuc produced a specific activity of 9.1 × 10^22^ photons s^−1^ mol^−1^, corresponding to a total activity of 2.5 × 10^13^ photons s^−1^ mL^−1^, using a bacterial expression system and a prokaryotic signal peptide for periplasmatic expression [40]. Welsh *et al.* reported the effective application of cell-free protein synthesis (CFPS) for the production of GLuc, possessing a specific activity of 9.0 × 10^23^ photons s^−1^ mol^−1^ and a half life of 1.3 (±0.3) min [38]. The short half-life of the *Gaussia* luciferase enzyme leads to the question whether it is enough to influence the MC to SP interconversion. Recently, GLuc mutants with a considerably prolonged half life of 13.5 (±4) min have been produced that possess a high specific activity of 8.1 × 10^23^ photons s^−1^ mol^−1^ [38]. Indeed, these values indicate that the amount of light produced by luciferase is in the range of the minimal total photon emission rate necessary to induce a conformational change of spiropyran-DO3A-Gd. 

In principle, two different possible mechanisms of excitation of a photosensitive molecule exist: Radiative absorption of the bioluminescence emission and direct energy transfer to the photosensitive molecule [41]. Theodossiou *et al.* and Carpenter *et al.* have shown that the overexpression of FLuc is capable to activate photosensitive molecules used as photosensitizers [41,42]. In preliminary experiments we could not observe a direct energy transfer of the Foerster type or radiative absorption after mixing coelenterazine, GLuc and spiropyran-DO3A-Gd (data not shown). Also an external illumination using various enzyme concentrations could not induce a MC to SP isomerization (data not shown). But improvements of the enzyme production and prolonging the half-life of GLuc could clearly lead to a MC to SP isomerization. We are currently working on improvements to the DO3A-tethered photochromic molecules to improve light sensitivity as well as mutations to the luciferases to improve light production and prolonging enzymatic half life. 

## 3. Experimental

Synthesis of spiropyran conjugated to the macrocyclic ligand 1,4,7,10-tetraazacyclododecane 1,4,7-triacetic acid (spiropyran-DO3A) was performed as previously reported [8]. For Gd(III) complexation, a mixture of spiropyran-DO3A (0.124 g) and Gd(OTf)_3_ (0.11 g) in methanol (6 mL) was stirred for 24 h at 60 °C. The solvent was evaporated *in vacuo*. The orange-colored solid powder was stored at room temperature in the dark for long-term storage. Prior to use, the powder was rehydrated in water (ultra-pure grade, Cellgro, Manassas, VA, USA) and stored at 4 °C for short term use. Solution was used for a maximum of 14 days after rehydration. Samples were prepared either in water or ethanol (Sigma-Aldrich, St. Louis, MO, USA). 

The crowned spiropyran and crowned spiropyran complexed with Gd(III) ions were dissolved in water and further diluted in water or 100% EtOH to 50.58 (±11.77) μM for spiropyran-DO3A and 44.32 (±6.09) μM for spiropyran-DO3A-Gd. This resulted in peak absorbance values between 0.6 to 0.5 for the aqueous solutions and 0.6 to 0.7 for the ethanol solutions.

**Figure 9:**
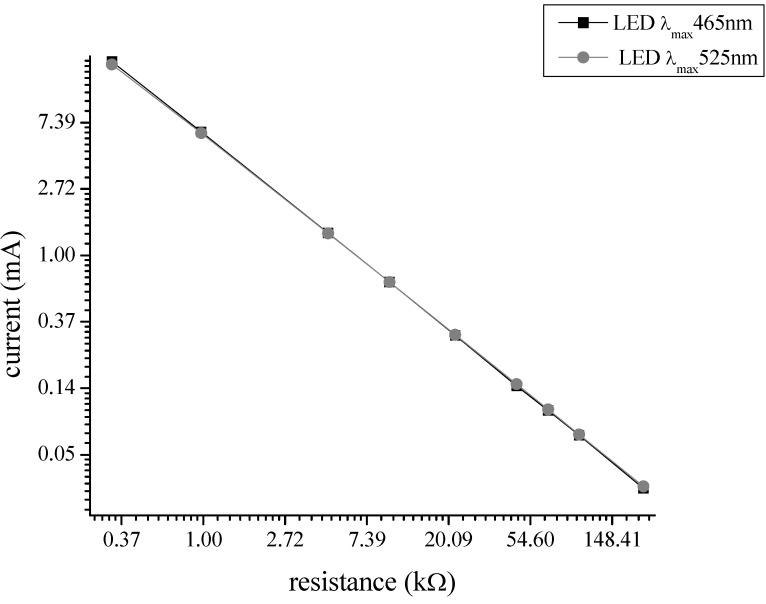
Determination of current: Different resistors between 330 Ω and 220 kΩ were connected with the LED in series. For experiments a 9 V voltage supply was used. The voltage drop at the resistor was measured and the resulting current calculated.

The UV-visible spectra were recorded with an UV-Vis spectrophotometer (Cary 100Bio) in quartz cells (Starna Cells, Atascadero, CA, USA, catalog number 18/9-Q-10) in 250 μL of solution. For the illumination experiments a blue LED (650 millicandela typical intensity, viewing angle of 154 degrees and maximal emission at 465 nm, RadioShack, Fort Worth, Texas, USA catalog number 276-013) and a green LED (960 millilumen typical intensity, viewing angle of 130 degrees and maximal emission at 525 nm, RadioShack catalog number 276-027) were used. The illuminance by the LED was adjusted with the constant current source. The current source was a 9 V regulated voltage source (TENMA, Newark, Palatine, IL, USA) and calibrated resistors placed in series with the LED to produce currents up to 18.5 mA (measured by Tektronix TX3 multimeter; Figure 9). The largest current produces no heating induced effects in the emission spectra. The spectral characteristics of the blue-emitting LED and the green emitting LED are displayed in Figure 8A. The relationship between the operating current and the photon rate is shown in Figure 8B. 

The LED has a rectangular package. The LED package was positioned next to the surface of the quartz cell, ensuring a maximum light illumination to the interior, fluid containing gap of the quartz cell. Irradiation was performed for defined time periods (1 s up to 10 min). The cuvette was briefly removed from the holder, placed in the UV-Vis spectrophotometer followed by UV-visible spectroscopic measurement. The emission spectra (between 400 nm and 650 nm) produced during the illumination period were recorded. For control conditions, quartz cells subjected to the same number of measurements without LED light illumination were performed. The kinetic rate constants for the light-induced isomerization from the merocyanine to the spiropyran form were extrapolated from the slope of the plot as described [43]:


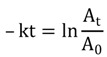


where A_t_ = peak absorption value at the time point t after illumination and A_0_ = peak absorption value before illumination The total LED power emitted was determined with a calibrated optical power meter (Newport Corp840 and sensor 818-SC). The LED was placed with minimal distance in front of the sensor to simulate comparable illumination conditions as used for the quartz cell illumination. Calibration of the power meter was performed at the maximum emission wavelengths of the LEDs (blue: 465 nm, green: 525 nm). The LED emission spectra was measured with an optical spectrum analyzer (Ando, now Yokogawa, AQ-6315A, Sugar Land, TX, USA) and then scaled to the total LED emission power to produce power distribution as a function of wavelength W_λ_. 

The photon emission rate was measured in the following manner: The photon emission rate per nanometer at a fixed current is:


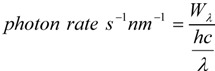


and the total photon rate emitted by the LED over the emission spectrum is: 


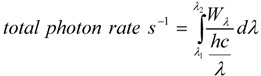


where h = 6.63 × 10^−34^ J·s (Planck’s constant, c = 3.00 × 10^8^ m·s^−1^ (velocity of light) and λ is in nm. Total amount of photons were determined using the integral of (2) from λ_1_ = 450 nm to λ_2_ = 650 nm (green LED) and from λ_1_ = 400 nm to λ_2_ = 554 nm (blue LED) (3).

The relationship between the diode current, electrical power and total photon emission rate for the calibrated resistors used in the current investigation is displayed in Table 2 for the blue LED and for the green LED:

**Table 2:** Illumination with two LEDs: Diode current, electrical power and total photon emission rate were determined for the blue emitting LED and the green emitting LED.

	Blue emitting LED		Green emitting LED	
Current (mA)	Total radiant flux (μW)	Photon emission rate (photons·s^−1^)	Total radiant flux (μW)	Photon emission rate (photons·s^−1^)
17.676	7536	1.7542 × 10^16^	2467.615	6.5372 × 10^15^
6.314	2927.58	6.8873 × 10^15^	1133.615	2.9919 × 10^15^
1.3978	588.72	1.3702 × 10^15^	298.405	7.9035 × 10^14^
0.6687	248.4	5.7821 × 10^14^	151.08	4.0141 × 10^14^
0.3031	82.884	1.9289 × 10^14^	66.3875	1.7577 × 10^14^
0.1443	26.334	6.1284 × 10^13^	29.29	7.7539 × 10^13^
0.0984	13.46	3.132 × 10^13^	18.93	5.0114 × 10^13^
0.0675	6.4596	1.5027 × 10^13^	12.15	3.2162 × 10^13^
0.0309	1.0182	2.37 × 10^13^	4.634	1.2267 × 10^13^

*T*_1_ measurements were performed at constant temperature of 37 °C with a relaxometer (Bruker minispec, mq60, Bruker Biospin, Rheinstetten, Germany). Adjustments for measuring *T*_1_ values for the determination of *r*_1_ were: First pulse separation = 30 ms; final pulse separation = 13,000 ms; number of data points used for fitting = 12; delay sampling window = 0.03 ms; sampling window = 0.015 ms; time for saturation curve display = 3 s; total analysis time = 12.16 min. Concentrations of spiropyran-DO3A-Gd between 39.4 μM and 157.6 μM were used in a total of 400 μL water in flat-bottom NMR-tubes with a diameter of 0.6 mm. The *r*_1_ values were determined after plotting 1/*T*_1_ values against the respective varying concentrations of the contrast agent, either kept in the dark or illuminated. Before the measurement, samples were adjusted to 37 °C. For the determination of ΔT_1_ values after illuminating with different photon emission rates and different time periods, a different measurement-sequence was used to reduce the analysis time from 12.16 min to 7.15 min. Additional parameters were: First pulse separation = 10 ms; final pulse separation = 10,000 ms; number of data points used for fitting = 12; delay sampling window = 0.031 ms; sampling window = 0.015 ms; time for saturation curve display = 3 s.

The 591 bp cDNA of humanized *Gaussia princeps* luciferase (NanoLight, Pinetop, AZ, USA) was amplified with Polymerase Chain Reaction (PCR) using primers (5'-ACTGATCCATGGGAGTCAA AGTTCTGTTTGC-3' and 5'-ACAGTACTCGAGAGCGGCCGCTTGGTCACCACC-3'). Through the introduction of a 5' *SalI* and 3'*NheI* restriction site, the gene was introduced in frame into the expression vector pET28b (Addgene, Cambridge, MA, USA), now bearing a 3'-terminal polyhistidine tag (5'-CAGCAGCAGCAGCAGCAG-3'). Subcloning was performed in NEB5-alpha bacterial strain and verification was performed with restriction analysis using *SacII* und *MluI* and DNA sequencing. As protein expression system, competent JM109 (DE3) cells were transformed with the plasmid. Overexpression was started at a OD_600 nm_ of 0.4 after induction with 1.0 M isopropyl β-D-1-thiogalactopyranoside (IPTG) and continuously stirred at 15 °C overnight. The bacterial culture was harvested at an OD_600 nm_ of 0.8 and centrifuged in a Sorvall RC-5BPlus centrifuge (Thermo Scientific, Asheville, NC, USA, USA) equipped with a GSA rotor at 2,700 ×*g*. Bacterial pellets were resuspended in Tris-buffered saline, pH = 7.6, sonicated and purified with a Protino Ni-IDA column (Macherey-Nagel, Bethlehem, PA, USA). Protein concentration was determined with the BCA assay (Pierce Biotechnology, Rockford, IL, USA) in a microplate reader (TecanSafire^2^, Tecan Group Ltd., Männedorf, Switzerland) at 562 nm. Validation of the product was performed with SDS-polyacrylamide electrophoresis (Bio-Rad, Hercules, CA, USA) and a 7–175 kDa marker (catalog-number P7709V, New England Biolabs, Ipswich, MA, USA) as reference. Emission spectra were recorded with the HORIBA Jobin Yvon Fluoro Max-P spectrophotometer after supplementation of coelenterazine (Wako Chemicals USA, Inc, Richmond, VA, USA). Coelenterazine stock solution was prepared in EtOH and further diluted in TBS buffer. Optical imaging was performed in an IVIS-100 system (Caliper Life Science, Hopkinton, MA, USA). Parameters used for measurements: Open filter, field of view 10, f-stop 16, exposure time 5 s and binning 4. Repetitive measurements were performed after adding the substrate with a time delay of approximately 25 s between each measurement. Protein lysates were placed into a white colored 96 multiwell polysterene plate (Nalge Nunc International, Rochester, NY, USA) prior measurement. We calculated the total photon rate (equivalent to the total flux) based on a region of interest (ROI) measurement. The radiance in each pixel was integrated over the ROI and multiplied with 4π. A ROI area of 0.419 (±1.89 × 10^−3^) cm^−2^ was used.

## 4. Conclusions

The work performed here describes, as a longterm goal, a method to noninvasively map gene expression in deep tissues *in vivo* by developing magnetic resonance imaging contrast agents (MRI CAs) that are responsive to commonly employed luminescent biomarker systems. The results reported here show promise that a reversible MRI CA that responds to luciferase is feasible. Moreover, using LEDs to mimic bioluminescence emission has proven to be a very effective model system to experimentally induce photochromic molecule isomerization and comparison with a biological system. Application of the spiropyran-DO3A-Gd CA will be of great utility to allow for deep tissue probing of gene expression using non-invasive imaging methods.
